# Exploring the Role of the Nurse Case Manager in the Care Experience of Patients Undergoing Radical Cystectomy: A Qualitative Study

**DOI:** 10.1002/nop2.70821

**Published:** 2026-09-29

**Authors:** Erika Acquistapace, Bruno Bernardini, Federico Piccioni, Manuela Pastore, Maria Vittoria Fantacci, Pietro Brin, Stefano Mancin, Roberto Contieri, Daniela Cattani, Simone Cosmai, Beatrice Mazzoleni, Rodolfo Hurle

**Affiliations:** ^1^ Humanitas University Pieve Emanuele, Milan Italy; ^2^ Neuro‐Rehabilitation Unit, Rehabilitation Department, Neuro‐Center IRCCS Humanitas Research Hospital Rozzano, Milan Italy; ^3^ Anesthesia and Perioperative Medicine Unit, Department of Anesthesia and Intensive Care IRCCS Humanitas Research Hospital Rozzano, Milan Italy; ^4^ Clinical Nutrition Unit IRCCS Humanitas Research Hospital Rozzano, Milan Italy; ^5^ Department of Urology IRCCS Humanitas Research Hospital Rozzano, Milan Italy; ^6^ Department of Biomedical Sciences Humanitas University Pieve Emanuele, Milan Italy; ^7^ Department of Urology Istituto Nazionale Tumori IRCCS Fondazione G. Pascale, IRCCS Naples Italy; ^8^ IRCCS Humanitas Research Hospital Rozzano, Milan Italy

**Keywords:** bladder cancer, frailty, Nurse Case Manager, qualitative research, radical cystectomy

## Abstract

**Aim:**

To explore patients' lived experiences of the Nurse Case Manager (NCM) within the GRACE‐M radical cystectomy pathway, focusing on relational support, care coordination, continuity and recovery‐related challenges.

**Design:**

A qualitative phenomenological study was conducted, following Giorgi's methodology.

**Methods:**

Qualitative data were collected through 16 semi‐structured interviews conducted 6 months after discharge with patients who had undergone elective RC within a multidisciplinary care pathway at a tertiary university hospital in Northern Italy. Data were analysed using Giorgi's phenomenological method to explore in depth patients' perceptions of the role of the NCM and their overall care experience.

**Results:**

Patients perceived the NCM as both a reassuring presence and an accessible care coordinator who facilitated information, specialist contact and continuity after discharge. No distinct NCM‐related barrier theme emerged; reported difficulties concerned post‐operative recovery and adaptation.

**Conclusion:**

Patients perceived the NCM as combining emotional support with coordination and navigation. Given the single‐centre qualitative design and interview self‐selection, these findings describe perceived contributions and do not demonstrate causal effects on satisfaction, adherence or clinical outcomes.

**Patient or Public Contribution:**

The data consists of answers given by patients in interviews.

## 1 Introduction

Bladder cancer represents one of the most prevalent malignancies in both the United States and Italy, with a particularly high incidence among elderly and male populations. In 2024, an estimated 83,190 new cases of bladder cancer are expected to be diagnosed in the United States, accounting for approximately 4.2% of all cancer diagnoses (Siegel et al. 2024). Similarly, in Italy, around 29,700 new cases were recorded in 2023, with men comprising the majority (about 23,700 cases) (AIOM 2023). The increasing incidence underscores the need for effective management strategies, particularly for muscle‐invasive bladder cancer (MIBC, t 2–4), where radical cystectomy remains the gold standard of treatment (Fischer et al. 2020). Preoperative chemotherapy is commonly employed to enhance survival outcomes and reduce the risk of recurrence (Fan et al. 2023).

In recent years, Italy has witnessed a growing adoption of minimally invasive surgical techniques, such as laparoscopy and robot‐assisted surgery, which offer potential benefits in terms of reduced postoperative complications, shorter hospital stays and improved patient recovery (Porreca et al. 2022). However, achieving optimal outcomes necessitates a comprehensive, multidisciplinary approach that takes into account crucial factors such as patient frailty, comorbidities and nutritional status, all of which play a pivotal role in the therapeutic pathway (Dal Bello et al. 2024; Rodrigues Pessoa et al. 2020). To address the complexities associated with radical cystectomy, the GRACE‐M (Global Radical Cystectomy Evaluation and Management) program was developed at a tertiary university hospital in Northern Italy. This innovative program aims to enhance patient outcomes by implementing a multidisciplinary, patient‐centered approach that integrates specialists from various fields including urology, oncology, neurogeriatrics, dietician and physiotherapy (Bernardini et al. 2025).

The GRACE‐M program consists of several key phases. The preoperative phase involves a comprehensive assessment of patient frailty and nutritional risk using validated screening tools and questionnaires. Specialist consultations are tailored to the patient's specific needs, focusing on optimizing physical and nutritional status before surgery. This phase leads to the development of a personalized surgical plan that considers individual risk factors and aims to minimize perioperative complications. During hospitalization, the Institutional Acute Pain Service Team implements a multimodal approach to postoperative pain management, focusing on minimizing opioid use and reducing medication‐related adverse effects. All patients are started on a regular diet on the day of surgery, unless contraindicated. Patients identified as being at risk for malnutrition are provided with high‐protein oral nutrition supplements. Early mobilization is facilitated by the physiotherapy team. The multidisciplinary team ensures adherence to perioperative protocols and ERAS (Enhanced Recovery After Surgery) guidelines. Interventions are provided to address emerging complications and optimize recovery. After discharge from the hospital, structured follow‐up assessments are performed at 15, 30 and 180 days and 1 year after surgery through multidisciplinary consultation among the specialists involved and integrating telephone consultations to monitor recovery progress, identify complications early, and provide rehabilitation guidance (Bernardini et al. 2025). A cornerstone of the GRACE‐M program is the integration of the Nurse Case Manager (NCM), a key figure responsible for coordinating the entire patient pathway. The NCM serves as the primary liaison between the patient and the multidisciplinary team, ensuring seamless communication, continuity of care and adherence to treatment protocols (Bakker et al. 2023). Their responsibilities include coordinating specialist consultations and facilitating communication among healthcare professionals, overseeing the management of frailty and nutritional interventions to optimize preoperative preparation, ensuring compliance with ERAS guidelines to enhance recovery and reduce complications and conducting structured follow‐ups to monitor patient well‐being and gather critical data to refine treatment strategies (Wang, Chen, et al. 2022; Wang, Huang, et al. 2022). By providing consistent support and personalized care, the NCM plays a crucial role in improving clinical outcomes, reducing hospital stay durations, minimizing readmission rates and ultimately enhancing the patient's quality of life (Chartrand et al. 2023). Additionally, in this context, a structured multidisciplinary approach enables healthcare providers to deliver holistic, patient‐centered care that addresses the complex needs of individuals undergoing radical cystectomy (Desideri et al. 2024). Evidence suggests that such comprehensive care models contribute to improved postoperative recovery, reduced morbidity and enhanced overall well‐being (Saripella et al. 2021). Moreover, the proactive identification and management of risk factors during the preoperative phase can significantly impact long‐term outcomes and healthcare resource utilization (Yao et al. 2024).

### 1.1 Aim

The objective of this study was to explore patients' lived experiences and perceptions of the NCM within the multidisciplinary GRACE‐M pathway. The analysis focused on the NCM's relational support, accessibility, communication, care coordination and navigation, continuity across settings, and the recovery‐related challenges described by patients. The study was not designed to quantify the NCM's effectiveness or to test a prespecified set of organizational barriers; barriers were considered only when they emerged from participants' narratives.

## 2 Methods

### 2.1 Study Design

This qualitative phenomenological study applied Giorgi's descriptive phenomenological methodology (1997), which explores the essence of lived experiences through the systematic analysis of participants' narratives. This approach was considered appropriate because the study aimed to understand how patients experienced the role of the Nurse Case Manager within a multidisciplinary radical cystectomy pathway.

According to Giorgi's method, the analysis involved four main steps. First, each interview transcript was read in its entirety to obtain a general sense of the whole experience described by the participants. Second, the transcripts were divided into meaning units whenever a relevant shift in meaning occurred in relation to the phenomenon under investigation. Third, these meaning units were transformed from participants' everyday language into nursing and scientific expressions, whereas preserving the original meaning of the narratives. Finally, the transformed meaning units were compared across interviews and synthesized into themes and subthemes representing the essential structure of the patients' experiences. The study adhered to the Consolidated Criteria for Reporting Qualitative Research (COREQ) (Tong et al. 2007) to ensure methodological rigour and transparency (Table S1).

### 2.2 Ethical Considerations

The study was conducted in compliance with good clinical practices, after obtaining ethical approval from redacted, and informed consent from all participants, obtained prior to enrollment in the study, in accordance with ICH regulations and in compliance with the current ‘Information and Consent to the Processing of Personal Data’ under Article 13 of EU Regulation 679/2016. Furthermore, the principal investigator ensured that the study was conducted in agreement with the Helsinki Declaration.

### 2.3 Sample, Criteria and Context

Participants in the qualitative component were recruited exclusively from patients already enrolled in the GRACE‐M program. At the study site, patients with a histological diagnosis of muscle‐invasive bladder cancer who were scheduled for elective radical cystectomy with curative intent and had sufficient time to complete the protocolized preoperative multidisciplinary assessment were eligible for GRACE‐M. Patients requiring urgent radical cystectomy because of uncontrollable haematuria, severe symptoms or an interval too short to complete the preoperative assessment were not eligible for the program. All 34 GRACE‐M participants who underwent elective radical cystectomy during the study period were considered for the 6‐month interview phase; 16 provided separate written consent and completed the interview. Thus, prior participation in GRACE‐M was a prerequisite for inclusion in this qualitative study, and patients treated outside the program were not sampled.

A total of 16 participants were included in the study. The sample size was deemed appropriate based on the principles of Giorgi's phenomenological method, which emphasizes the depth and richness of qualitative data over numerical representativeness. The chosen sample size aligned with the methodological approach, which seeks to achieve a comprehensive understanding of the lived experiences of participants rather than generalizability. Furthermore, the number of participants was determined by the principle of data saturation, wherein additional interviews would not yield new or meaningful insights relevant to the research objectives (Giorgi 1997).

### 2.4 Study Procedure

This study was embedded within the GRACE‐M program (Bernardini et al. 2025) and examined patients' perceptions of the NCM within the multidisciplinary pathway for elective curative radical cystectomy. The NCM entered the pathway during the preoperative phase and remained the designated nursing point of contact through hospital admission, surgery, discharge, and scheduled follow‐up. Within the program, the NCM coordinated multidisciplinary appointments and referrals, facilitated communication among professionals, consolidated information for the patient, monitored planned pathway milestones and nursing care needs, supported preparation for discharge, and escalated emerging concerns to the relevant specialist. Follow‐up telephone contacts were scheduled at 15, 30 and 180 days and 1 year after discharge. Patients also received a dedicated email address for non‐urgent requests and a telephone number for urgent situations. These pathway‐defined functions are represented in Figure 1 and are distinguished from the patient‐reported functions presented in the Results.

**FIGURE 1:**
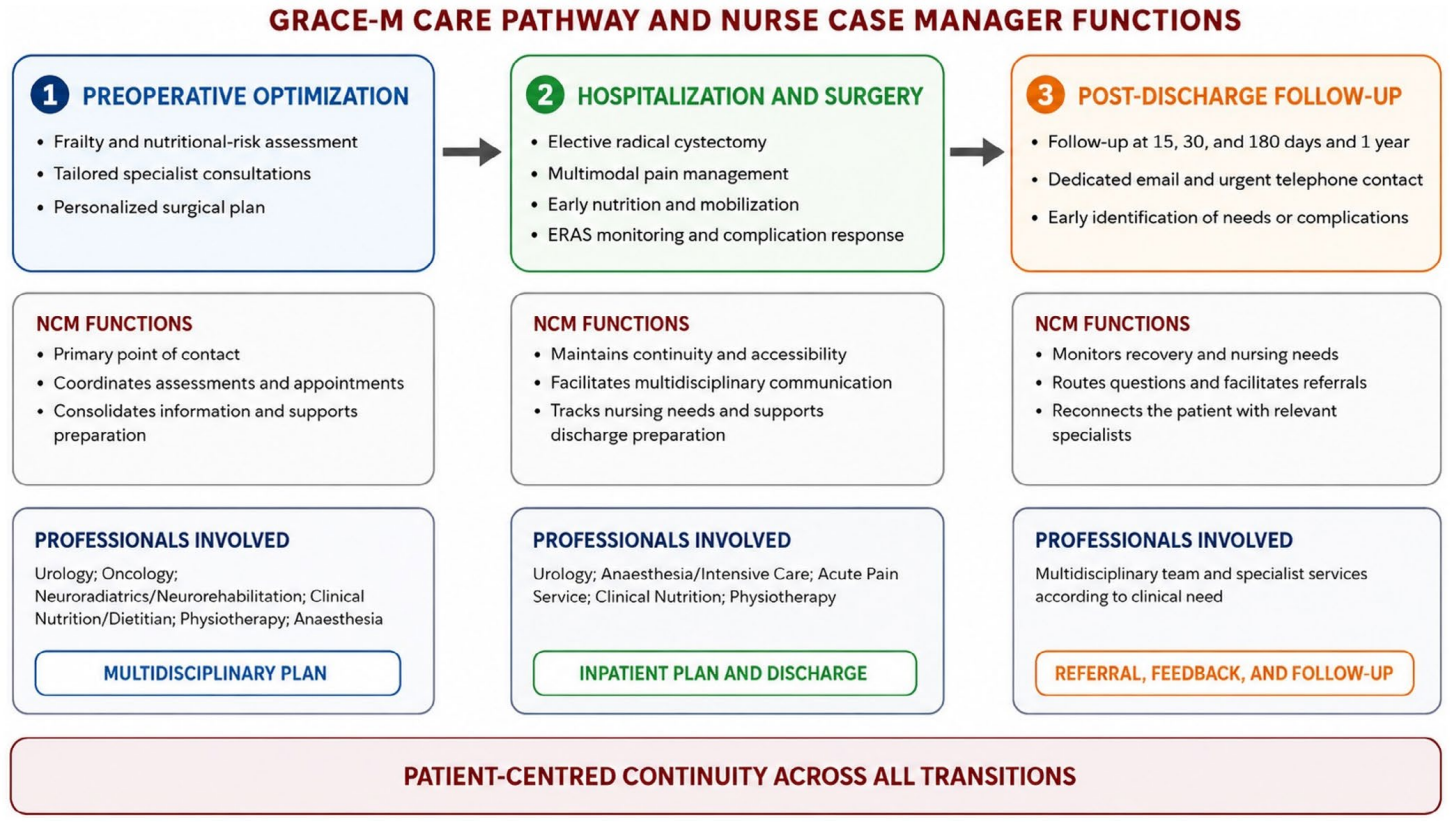
GRACE‐M multidisciplinary care pathway and Nurse Case Manager functions. ERAS, Enhanced Recovery After Surgery; GRACE‐M, Global Radical Cystectomy Evaluation and Management; NCM, Nurse Case Manager. Professional involvement is tailored to individual patient needs.

Phenomenological data were collected regarding patients' perceptions and experiences, following Giorgi's phenomenological method, which is based on a deep and rigorous understanding of subjective experiences while maintaining a degree of objectivity in the analysis process (Giorgi 1997). The researcher (E.A.) conducted the interviews digitally via Microsoft Teams, between September and October 2024 to assess patients' opinions regarding the role of the NCM in their care pathway. To fully capture patients' experiences and emotions, the interviews were scheduled 6 months post‐discharge. This timing was chosen to allow participants sufficient time to complete the early recovery phase, experience post‐discharge follow‐up, and reflect on the role of the NCM throughout the care pathway. It was considered appropriate to capture both immediate and medium‐term perceptions of recovery, continuity of care and emotional support. This timeframe was determined based on a multidisciplinary consultation among healthcare professionals involved in the GRACE‐M program. The interviews were conducted in Italian, lasted a maximum of 30 min, and took place in a virtual environment to ensure an adequate level of comfort that encouraged open and non‐judgmental communication (Table 1). Each interview was recorded and subsequently transcribed verbatim to capture the richness of the dialogue.

**TABLE 1:** Interview guide for scheduled interviews.

(1) How would you define your health status now compared to when you started the care pathway in the GRACE‐M study?
(2) The NCM accompanied you throughout the entire process: How did you perceive their presence? Could you describe a specific situation in which the Case Manager's role was particularly relevant to you?

Abbreviations: GRACE‐M, Global Radical Cystectomy Evaluation and Management; NCM, Nurse Case Manager.

The interviews were conducted by E.A., a researcher with experience in qualitative data collection and familiarity with the GRACE‐M care pathway. Before data collection, the interviewer reflected on her professional background and potential assumptions regarding the NCM role. No therapeutic or hierarchical relationship existed between the interviewer and the participants, who were informed about the study aims, the interviewer's role, and the voluntary nature of participation. During the interviews, a neutral and non‐judgmental attitude was adopted to encourage participants to freely describe both positive and negative experiences. Member checking was not formally conducted; however, clarification and summarizing strategies were used during the interviews when necessary to verify the meaning of participants' statements.

Data analysis was performed by two researchers (S.M., E.A.) according to Giorgi's descriptive phenomenological method. To strengthen confirmability, both researchers independently completed the analytical sequence for all transcripts including repeated reading, identification of meaning units, transformation of participants' everyday expressions into nursing and scientific language and preliminary grouping into subthemes and themes. E.A. had conducted the interviews, whereas S.M. had not participated in data collection and therefore provided an independent analytical perspective. The two researchers then compared their analytical matrices and proposed categorizations. Any discrepancy was discussed, the relevant original transcript was revisited, and a consensual formulation was documented before the final thematic synthesis was agreed.

Methodological rigour was supported by applying the criteria of credibility, transferability, dependability and confirmability. Credibility was enhanced through verbatim transcription, independent analysis by two researchers, consensus discussions and representative quotations. Transferability was supported by detailed reporting of the study setting, the GRACE‐M pathway, participant characteristics and interview procedures. Dependability was supported by an audit trail documenting meaning‐unit segmentation, transformations, coding decisions, theme development and consensus discussions. Confirmability was supported by reflexive notes, the independent full‐dataset review described above, and repeated comparison of interpretations with the original transcripts. Member checking and an external third‐party phenomenological audit were not performed. Data saturation was assessed jointly during analysis and was considered reached when additional interviews generated no new meaning units, subthemes or insights relevant to the study aim. The final synthesis comprised the five main themes and related subthemes reported in Table 3.

## 3 Results

### 3.1 Study Sample

Thirty‐four patients who underwent elective radical cystectomy for curative purposes were enrolled in the GRACE‐M program and were eligible for invitation to the qualitative component. Sixteen provided separate consent and completed the 6‐month interview, corresponding to a participation rate of 47.1%. The other 18 patients did not complete the interview because consent was not provided and/or they were unavailable for the scheduled interview; separate counts for these two reasons were not recorded (Figure 2).

**FIGURE 2:**
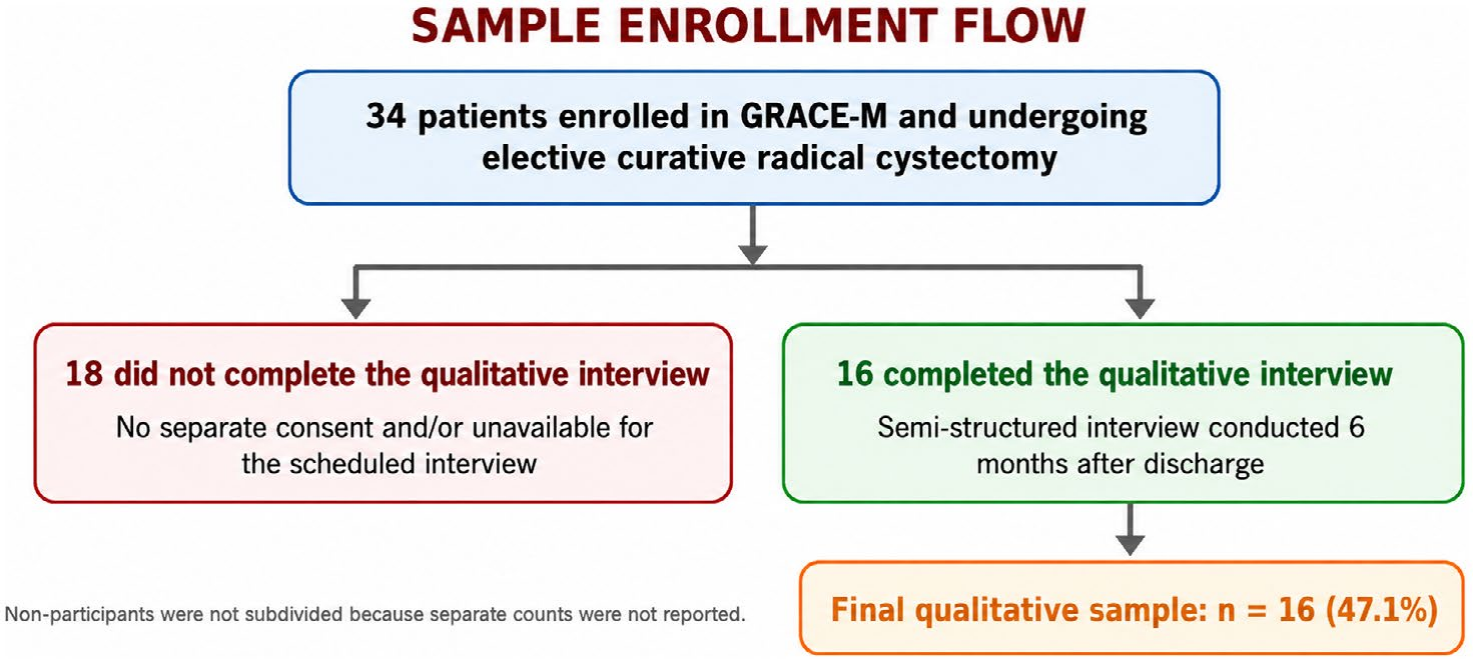
Sample enrollment flowchart. GRACE‐M, Global Radical Cystectomy Evaluation and Management.

Because the qualitative interviews were conducted only among patients who agreed to participate, a potential self‐selection bias cannot be excluded. Participants may have been more willing to share their experiences, more engaged with the care pathway, or more satisfied with the support received from the NCM than those who did not participate. A formal comparison between participants and non‐participants was not performed because detailed demographic and clinical variables for non‐participants were not systematically collected for the qualitative component of the study. To provide a more comprehensive description of the study sample, the demographic and clinical characteristics of the interviewed participants are reported in Table 2.

**TABLE 2:** Participant characteristics.

Characteristic	Category/statistic	Baseline (*n* = 34)	Participants (*n* = 16)
Participants		34 (100.0)	16 (100.0)
Sex	Male	29 (85.3)^a^	13 (81.3)
	Female	5 (14.7)	3 (18.7)
Age, years	Mean (SD)	66.7 (10.8)	69.9 (11.8)
	Median (IQR)	70 (19)	74.5 (57–79)
	Range	—	53–83
Age group	50–59 years	—	6 (37.5)
	60–69 years	—	2 (12.5)
	70–79 years	—	5 (31.3)
	> 80 years	—	3 (18.7)
BMI	Mean (SD)	27.6 (5.2)	—
	Median (IQR)	26.9 (8.2)	—
NRS 2002	Mean (SD)	1.3 (1.6)	—
	Median (IQR)	0.0 (3)	—
Active smokers		10 (29.4)	—
History of previous bladder cancer		9 (26.5)	—
High‐grade bladder cancer		34 (100.0)	—
Neoadjuvant treatment		8 (23.5)	—
Robotic surgery		3 (8.8)	—
Post‐operative intensive care		2 (5.9)	—
Discharge destination	Home	25 (73.5)	—
	Home with ADI	1 (2.9)	—
	Long‐term care facility	2 (5.9)	—
	Rehabilitation facility	2 (5.9)	—

*Note:* Values are reported as *n* (%) unless otherwise specified. Missing data are indicated by ‘—’.

Abbreviations: ADI, home nursing care; BMI, body mass index; IQR, interquartile range; NRS 2002, Nutritional Risk Screening 2002; SD, standard deviation.

^a^ Male count and percentage for Cohort 2 were derived from total participants (*n* = 34) minus reported females (*n* = 5).

### 3.2 Qualitative Results

Two researchers (S.M., E.A.) independently analysed the interviews, classifying the concepts that emerged into distinct aspects. To ensure methodological rigour and validity of the results, the criteria of credibility, transferability, dependability and confirmability were applied, as defined in a previous study (Johnson et al. 2020).

The interview excerpts were reported and coded with the notation ‘P*n*’, where ‘P’ represents the patient and ‘*n*’ corresponds to the number assigned to the patient during the data analysis phase (Table S2). The results of the interviews were then categorized into five main themes with their corresponding subthemes. The five main themes identified from the phenomenological analysis of the qualitative interviews were: (1) Post‐Operative Health Status; (2) Role of the NCM; (3) Overall Experience of Hospitalization and Recovery; (4) Perception of the Case Manager and (5) Emotional Impact of the Care Path (Table 3).

**TABLE 3:** Themes and subthemes from qualitative interviews.

Main themes	Subthemes	Patients
Post‐Operative Health Status	Physical Improvement	P1, P5
	Persistent Physical Difficulties	P7, P10
Role of the Case Manager	Emotional and Psychological Support	P3, P16
	Accessibility, Care Coordination and Navigation	P10–P12, P16
Overall Experience of Hospitalization and Recovery	Management of Complications	P10, P16
	Continuity of Care	P10
Perception of the Case Manager	Professionalism and Humanity	P9, P11
	Reassurance and Anxiety Management	P14, P16
Emotional Impact of the Care Path	Moral Support During Hospitalization	P3, P4
	Patient‐NCM Relationship as a Trust Point	P6, P15

Abbreviation: P, patient.

No distinct theme concerning barriers to contacting or interacting with the NCM emerged from the phenomenological analysis. The difficulties described by participants related primarily to post‐operative recovery, adaptation to urinary diversion and complications rather than to the NCM's accessibility or coordination. This absence is reported descriptively and does not establish that organizational barriers were absent.

### 3.3 Theme 1: Post‐Operative Health Status

From the conducted interviews, two patients (P1, P5) provided detailed descriptions of their physical progress after the surgery. Specifically, many patients reported significant improvements in their health, highlighting progressive recovery. For instance, P1 stated:

“I am stronger now, whereas I was weaker before,” while P5 said: “My health has improved.”

Although most participants described the NCM positively, the interviews also revealed several challenges related to the post‐operative recovery process (P7; P10). These challenges mainly concerned persistent physical difficulties, adaptation to the urinary diversion, intestinal function recovery, weight loss and the occurrence of post‐operative complications. For example, some participants reported difficulties in accepting the use of bags, problems related to intestinal function, and the need to adapt to a new body condition:

“I still don't accept the bags,” or P10 reported, “Intestinal function takes time to recover.”

These findings suggest that, although the NCM was generally perceived as a supportive and reassuring figure, patients continued to experience relevant physical and emotional challenges after discharge.

### 3.4 Theme 2: The Role of the Nurse Case Manager: Relational Support and Care Coordination

Participants described the NCM through two interconnected dimensions. The first was relational and emotional: the NCM was perceived as a reassuring, approachable, and psychologically supportive presence (P3, P16):

“An extraordinary person, a friend.” (P3)

“They supported me especially from a psychological point of view.” (P16)

Beyond relational support, participants described the NCM through concrete coordination and navigation activities. P12 valued being able to contact the NCM after discharge; P11 reported that the NCM facilitated contact with an infectious disease specialist; P10 described the NCM as gathering information and acting as a bridge between the patient and the healthcare system; and P16 valued clear explanations that reduced uncertainty. Taken together, these accounts portray the NCM as an accessible point of contact who routed questions, mobilized specialist input, translated information, and maintained continuity across hospital and post‐discharge care: ‘I reached out to her even after discharge’ (P12) and ‘She put me in contact with the infectious disease specialist for treatment’ (P11).

Participants did not spontaneously describe medication reconciliation, formal training of family caregivers or coordination with social‐care resources as distinct experiences. These functions were therefore not attributed to the NCM as patient‐derived findings. Their absence from the interviews may reflect the scope of the interview guide or limited salience to participants rather than the absence of such activities within clinical practice.

### 3.5 Theme 3: Overall Experience of Hospitalization and Recovery

Post‐operative recovery was experienced heterogeneously, and participants who encountered complications described a particular need for timely and understandable information (P10, P16). P10 referred to post‐operative hospital visits for minor complications and to the NCM's help in gathering information, whereas P16 emphasized the clarity and precision of explanations about the surgery:

“The Nurse Case Manager explained everything to me, reassuring me a lot.” (P16)

Continuity of care was experienced as remaining connected to the care team across stages and settings rather than as an isolated interaction. P10 summarized this organizational function by stating: ‘She acts as a bridge between the patient and the healthcare system’.

### 3.6 Theme 4: Perception of the Case Manager

Patients' perceptions of the NCM were characterized by a combination of professionalism and humanity (P9, P11). Specifically, several patients described the NCM as highly professional, yet also empathetic and human, stating:

“The Nurse Case Manager is a delightful, well‐prepared person.” (P9)

“Quick and precise: a gem.” (P11)

The Case Manager's ability to instill calm during moments of uncertainty was particularly appreciated by patients. P14 highlighted how important the constant reassurance was, often through updates like: ‘I spoke with the urologist,’ which helped reduce anxiety. P16 also emphasized the value of the Case Manager's support, who, by clearly explaining alternatives to the surgery, significantly alleviated her concerns.

### 3.7 Theme 5: Emotional Impact of the Care Path

The support provided by the NCM had a profound emotional impact on the patients. Many (P3, P4) recognized that the Case Manager's presence in the hospital positively affected their morale:

“Her presence was very helpful.” (P3)

“She was important when I went from being operated on overnight.” (P4)

Furthermore, the relationship between the patients and the NCM developed into one of deep trust and closeness:

“I quickly became attached to her.” (P6)

Similarly, P15 acknowledged the personal involvement of the NCM, stating: ‘She didn't give me the impression of someone doing it just for a job; she was my point of reference’.

These bonds represented a key element in the care experience, providing patients with emotional support that went beyond clinical assistance.

## 4 Discussion

This qualitative study explored patients' perceptions and lived experiences within the GRACE‐M pathway; it did not measure the effectiveness or causal impact of the NCM. Participants described the NCM as both a relational resource and an organizational point of continuity, whereas also reporting the substantial physical and emotional complexity of recovery after radical cystectomy. These findings therefore clarify the perceived contribution of the NCM without implying that the NCM alone produced the reported recovery experiences. Despite the predominantly positive perception of the NCM, the findings also highlighted important challenges experienced by patients during the recovery process. Some participants reported persistent difficulties related to intestinal function, adaptation to the urinary diversion, body image changes, weight loss and post‐operative complications. These aspects indicate that the presence of the NCM does not eliminate the complexity of recovery after radical cystectomy, but may help patients manage uncertainty, access information, and feel supported during a demanding care pathway. Therefore, future improvements to the GRACE‐M pathway could include more structured educational interventions on stoma management, additional psychological support for body image adaptation, and reinforced post‐discharge monitoring for patients experiencing persistent symptoms or complications. In particular, the existing literature emphasizes the importance of ongoing monitoring and support during postoperative recovery, especially after high‐impact procedures such as radical cystectomy (Wessels et al. 2020). This support includes clinical interventions, such as pain management and prevention of complications, as well as psychological support aimed at promoting patients' sense of integrity and empowerment during recovery (Akortiakuma et al. 2022). Progressive improvement in physical health after surgery is generally considered an indicator of successful recovery (Blöndal et al. 2022). However, persistent difficulties should also be considered. Previous studies have shown that patients undergoing major abdominal or intestinal surgery may continue to experience difficulties in accepting physical changes, including stoma‐related changes, with consequences for psychological well‐being and body acceptance (Stavropoulou et al. 2021). Similarly, post‐operative pain and functional limitations may negatively affect quality of life (Mateu Arrom et al. 2019).

Within this context, our findings are consistent with literature describing case management as a combination of assessment, care planning, education, coordination, referral, monitoring and follow‐up (Karam et al. 2021; Wang, Chen, et al. 2022; Wang, Huang, et al. 2022; Garnett et al. 2020; Molina‐Gil et al. 2024; Martella et al. 2026). The patient narratives in the present study directly support a more specific subset of these functions: accessibility after discharge, consolidation of information, facilitation of specialist contact, communication across the multidisciplinary team, and continuity between care phases. They do not provide sufficient evidence to claim that participants experienced every formal case‐management competency such as medication reconciliation, structured family‐caregiver training or coordination with social‐care resources. This distinction was retained to avoid extending the Results beyond what the interviews support.

These findings are also aligned with oncology nursing literature, which emphasizes the central role of nurses in maintaining quality across the cancer care continuum. Previous qualitative evidence showed that cancer survivors and oncology professionals identify ongoing information, shared decision‐making, emotional support, and continuity of care as core components of high‐quality cancer care, and that oncology nurses may serve as care managers throughout the continuum of care (Bashkin et al. 2023). In line with this literature, the NCM in our study was perceived as a bridge between patients and the healthcare system, helping patients navigate post‐operative recovery, access information and maintain contact with healthcare professionals after discharge.

Our results also extend previous evidence by highlighting the relational dimension of the NCM role in the context of radical cystectomy. Patients did not describe the NCM only as an organizational figure, but also as a professional able to combine clinical competence, accessibility, empathy and reassurance. This finding is consistent with literature emphasizing that empathy and professional communication are essential for building trust and improving patients' care experiences (Höglander et al. 2023; Moudatsou et al. 2020). However, rather than interpreting emotional support and continuity of care as separate aspects, our findings suggest that they are closely interconnected: continuous accessibility to a trusted professional may itself contribute to reassurance, anxiety reduction, and a greater sense of safety during a complex recovery process.

The absence of a distinct NCM‐related barrier theme should not be interpreted as evidence that barriers were absent. The interview guide invited participants to describe the NCM's presence and particularly relevant situations but did not systematically probe each organizational competency or potential obstacle. Future qualitative studies should therefore include explicit prompts on access to the NCM, appointment and referral management, self‐care education, treatment monitoring, caregiver support and coordination with community and social resources.

In our sample, only two patients (P14 and P9) had undergone neoadjuvant chemotherapy prior to surgery. Due to the small number of patients, it was not possible to conduct an in‐depth evaluation of the impact of neoadjuvant chemotherapy in the analysed context. However, the experience reported by patient P14 provides useful insight into the perception of support received during the therapeutic journey. In particular, P14 acknowledged the importance of the Case Manager in providing reassurance and regular updates, such as ‘I spoke with the urologist’, which were perceived as helpful in reducing uncertainty. This suggests that, in an onco‐surgical setting, structured communication between patients and the multidisciplinary team may play an important role in supporting emotional adaptation and perceived continuity of care.

Overall, the findings support further evaluation of the NCM within multidisciplinary surgical oncology pathways. Participants' accounts suggest perceived value in care coordination, provision and clarification of information, facilitation of specialist access and post‐discharge continuity. Future research should prospectively define the NCM intervention, distinguish pathway‐defined functions from functions recognized by patients, and examine how structured discharge planning, remote monitoring and proactive follow‐up relate to recovery experience and continuity of care (Chan et al. 2023; Hyeon et al. 2023; Assolari et al. 2024).

### 4.1 Limitations and Future Perspectives

This study has some significant limitations, first and foremost the qualitative nature of the study, which reduces the generalizability of the data due to the variability of personal perceptions linked to individual, cultural and socio‐economic factors. Moreover, only 16 of the 34 eligible patients participated in the qualitative interviews. This may have introduced selection bias, as patients who agreed to participate may have differed from non‐participants in terms of motivation, willingness to discuss their care experience, relationship with healthcare professionals, or overall satisfaction with the GRACE‐M pathway. In particular, patients with more positive experiences may have been more likely to participate, potentially contributing to the predominance of favourable perceptions of the NCM reported in the findings.

No formal comparison between participants and non‐participants was conducted because detailed demographic and clinical information for non‐participants was not systematically collected within the qualitative interview dataset. Therefore, possible differences between the two groups could not be fully assessed. Future studies should prospectively collect basic demographic and clinical data for both participants and non‐participants in order to evaluate potential differences and better estimate the impact of selection bias.

Moreover, the follow‐up duration might have been insufficient to assess the evolution of patients' experiences in the long term, particularly regarding psychological adaptation and quality of life.

A further limitation concerns the interview guide, which did not explicitly explore every clinical and organizational competency commonly associated with nursing case management. Consequently, the absence of specific findings on medication reconciliation, structured self‐care education, family‐caregiver training, appointment management or coordination with social resources cannot be interpreted as evidence that these activities were not performed or were not important. More targeted questioning is required to evaluate these domains.

Although this study was conducted within the GRACE‐M program and in a single tertiary university hospital, some findings may be transferable to other complex surgical and oncology pathways, particularly major abdominal surgery such as pancreatic surgery, hepatobiliary surgery, colorectal cancer surgery and other high‐complexity abdominal operations. In these settings, patients often present with preoperative frailty, sarcopenia, nutritional risk or malnutrition, making early identification and optimization essential before surgery (Yang et al. 2024; Wang et al. 2023; Argillander et al. 2022). The perceived value of the NCM may therefore extend beyond radical cystectomy. By coordinating multidisciplinary assessment, supporting nutritional and frailty‐related interventions, promoting adherence to ERAS‐based pathways, and ensuring continuity of care after discharge, the NCM may help address the complex needs of frail or nutritionally vulnerable patients undergoing high‐complexity abdominal oncological procedures.

However, transferability should be interpreted with caution, as the organization of nursing roles, availability of case management services, multidisciplinary resources and follow‐up models may differ across institutions and healthcare systems. Future multicenter studies involving different hospitals, surgical pathways and organizational models are needed to assess whether the role and perceived benefits of the NCM observed in the GRACE‐M program can be replicated in other contexts. Further research should also explore how local resources, staffing models and patient characteristics influence the implementation and effectiveness of NCM‐led care coordination.

Therefore, these limitations highlight the need for further research, including larger and more diverse samples, integrating patients from different cultural and socio‐economic backgrounds. Additionally, multicentric studies could assess the replicability of the results, whereas longitudinal research would allow for the analysis of the evolution of perceived benefits and provide insights for optimizing multidisciplinary programs.

## 5 Conclusion

This qualitative study clarifies how interviewed patients experienced the NCM within the GRACE‐M radical cystectomy pathway. The NCM was perceived as a trusted and accessible professional who combined emotional reassurance with concrete coordination and navigation functions including information gathering, facilitation of specialist contact and continuity after discharge. These findings describe perceived contributions rather than a causal effect and do not establish that all formal case‐management competencies were experienced by participants. Larger, multi‐centre, and more explicitly focused qualitative studies are needed to examine the full clinical and organizational scope of the role and to identify potential barriers across different care settings.

## Author Contributions

**Erika Acquistapace:** investigation, methodology, conceptualization, writing – review and editing, writing – original draft, formal analysis. **Bruno Bernardini:** methodology, writing – review and editing, investigation, visualization. **Federico Piccioni:** investigation, methodology, supervision. **Manuela Pastore:** writing – review and editing, conceptualization, methodology, investigation. **Maria Vittoria Fantacci:** conceptualization, methodology, writing – review and editing, investigation. **Pietro Brin:** writing – review and editing, visualization. **Stefano Mancin:** conceptualization, methodology, writing – review and editing, investigation, supervision, formal analysis, software, data curation, funding acquisition. **Roberto Contieri:** writing – review and editing, visualization. **Daniela Cattani:** writing – review and editing, visualization. **Simone Cosmai:** writing – review and editing, visualization. **Beatrice Mazzoleni:** writing – review and editing, visualization, supervision, validation. **Rodolfo Hurle:** writing – review and editing, investigation, supervision, validation.

## Disclosure

Artificial Intelligence (AI) Use Declaration: Generative AI Statement: During the preparation of this work, the authors used ChatGPT (OpenAI, San Francisco, CA, USA) solely to improve the language and enhance readability. Following its use, the authors carefully reviewed and edited the content as necessary and take full responsibility for the final version of the publication.

## Funding

The publication fee for this work was covered by the Italian Ministry of Health’s “Ricerca Corrente” funding to the IRCCS Humanitas Research Hospital.

## Ethics Statement

The study was conducted in compliance with good clinical practices, after obtaining ethical approval from the Ethics Committee of the IRCCS Humanitas Research Hospital, Rozzano, Italy (protocol no. ICH‐020 V1.00 08/08/2022), and informed consent from all participants, obtained prior to enrollment in the study, in accordance with ICH regulations and in compliance with the current ‘Information and Consent to the Processing of Personal Data’ under Article 13 of EU Regulation 679/2016.

## Conflicts of Interest

The authors declare no conflicts of interest.

## Supporting information


**Table S1:** COREQ checklist.
**Table S2:** Transcription of phenomenological interviews.

## Data Availability

The data that supports the findings of this study are available in Supporting Information of this article.
