# Supplementary material for: Exploring the Role of the Nurse Case Manager in the Care Experience of Patients Undergoing Radical Cystectomy: A Qualitative Study
Source: Nurs Open. 2026 Sep 29;13(9):e70821. doi: 10.1002/nop2.70821 (PMC13621235; doi:10.1002/nop2.70821)
Supplement: Supplementary file 1 [file NOP2-13-e70821-s001.docx]

**Supplementary File**

**Table S1. COREQ Checklist**

Developed from:

Tong A, Sainsbury P, Craig J. Consolidated criteria for reporting qualitative research (COREQ): a 32-item checklist for interviews and focus groups. International Journal for Quality in Health Care. 2007. Volume 19, Number 6: pp. 349 – 357

| **Item No** | | **Guide Questions/Description** | **Reported on Page #** |  |  |  |
| --- | --- | --- | --- | --- | --- | --- |
| **Domain 1: Research team and reflexivity** | | | | |  |  |
| **Personal Characteristics** | | | | |  |  |
| 1. Interviewer/ facilitator | | Which author/s conducted the interview or focus group? | p. 5-6, Section 2.5: EA conducted the interviews. |  |  |  |
| 2. Credentials | | What were the researcher’s credentials? E.g., PhD, MD | E.A MScN  S.M PhD |  |  |  |
| 3. Occupation | | What was their occupation at the time of the study? | E.A RN  S.M Nurse/ Researcher |  |  |  |
| 4. Gender | | Was the researcher male or female? | p. 6, Section 2.5: interviewer referred to as “her”; consider stating gender explicitly. |  |  |  |
| 5. Experience and training | | What experience or training did the researcher have? | p. 6, Section 2.5: experience in qualitative data collection and familiarity with the care pathway. |  |  |  |
| **Relationship with participants** | | | | |  |  |
| 6. Relationship established | | Was a relationship established prior to study commencement? | p. 6, Section 2.5: no therapeutic or hierarchical relationship existed before interviews. |  |  |  |
| 7. Participant knowledge of the interviewer | | What did the participants know about the researcher? e.g. personal goals, reasons for doing the research? | p. 6, Section 2.5: participants were informed about aims, interviewer role, and voluntary participation. |  |  |  |
| 8. Interviewer characteristics | | What characteristics were reported about the interviewer/facilitator? e.g. Bias, assumptions, reasons and interests in the research topic | p. 6, Section 2.5: reflexivity, professional background, and assumptions regarding NCM role described. |  |  |  |
|  | **Domain 2: study design** | | | |  |  |
|  | **Theoretical framework** | | | |  |  |
|  | 9. Methodological orientation and Theory | What methodological orientation was stated to underpin the study? e.g. grounded theory, discourse analysis, ethnography, phenomenology, content analysis | p. 4-6, Sections 2.1 and 2.5: Giorgi descriptive phenomenological method. |  |  |  |
|  | **Participant selection** | | | |  |  |
|  | 10. Sampling | How were participants selected? e.g., purposive, convenience, consecutive, snowball | p. 4-5, Section 2.3: all eligible patients with MIBC who were candidates for RC and provided consent. |  |  |  |
|  | 11. Method of approach | How were participants approached? e.g., face-to-face, telephone, mail, email | p. 4-5, Sections 2.2-2.3: informed consent obtained before enrollment; exact approach method not fully specified. |  |  |  |
|  | 12. Sample size | How many participants were in the study? | p. 5 and p. 7, Sections 2.3 and 3.1: 16 participants. |  |  |  |
|  | 13. Non-participation Setting | How many people refused to participate or dropped out? Reasons? | p. 7, Section 3.1: 18 eligible patients did not participate because they did not consent or were unavailable. |  |  |  |
|  | 14. Setting of data collection | Where was the data collected? e.g., home, clinic, workplace | p. 5, Section 2.5: interviews conducted digitally via Microsoft Teams in a virtual environment. |  |  |  |
|  | 15. Presence of nonparticipants | Was anyone else present besides the participants and researchers? | Participants were generally interviewed alone; when necessary, a caregiver was present to support communication or participation. |  |  |  |
|  | 16. Description of sample | What are the important characteristics of the sample? e.g. demographic data, date | p. 7, Section 3.1 and Table 2: demographic and clinical characteristics reported. |  |  |  |
|  | **Data collection** | | | |  | No |
|  | 17. Interview guide | Were questions, prompts, and guides provided by the authors? Was it pilot tested? | p. 6-7, Section 2.5 and Table 1: interview guide reported. Pilot testing not reported. |  |  |  |
|  | 18. Repeat interviews | Were repeat interviews carried out? If yes, how many? | No. No repeat interviews were conducted. Each participant was interviewed once. |  |  |  |
|  | 19. Audio/visual recording | Did the research use audio or visual recording to collect the data? | p. 5, Section 2.5: each interview was recorded and transcribed verbatim. |  |  |  |
|  | 20. Field notes | Were field notes made during and/or after the interview or focus group? | p. 6, Section 2.5: field notes and reflexive notes maintained. |  |  |  |
|  | 21. Duration | What was the duration of the interviews or focus group? | p. 5, Section 2.5: interviews lasted a maximum of 30 minutes. |  |  |  |
|  | 22. Data saturation | Was data saturation discussed? | p. 6, Section 2.5: saturation determined when no new meaning units, subthemes, or relevant insights emerged. |  |  |  |
|  | 23. Transcripts returned | Were transcripts returned to participants for comment and/or correction? | p. 6, Section 2.5: transcripts were not formally returned; member checking not formally conducted. |  |  |  |
|  | **Domain 3: analysis and findings** | | | |  |  |
|  | **Data analysis** | | | |  |  |
|  | 24. Number of data coders | How many data coders coded the data? | p. 6, Section 2.5: two researchers (SM, EA) analyzed the data. |  |  |  |
|  | 25. Description of the coding tree | Did the authors provide a description of the coding tree? | p. 6 and p. 8, Section 2.5 and Table 3: meaning units, subthemes, and themes described. |  |  |  |
|  | 26. Derivation of themes | Were themes identified in advance or derived from the data? | p. 4-6, Sections 2.1 and 2.5: themes derived from participants’ narratives through Giorgi’s method. |  |  |  |
|  | 27. Software | What software, if applicable, was used to manage the data? | No qualitative data analysis software was used; transcripts were analyzed manually by the researchers. |  |  |  |
|  | 28. Participant checking | Did participants provide feedback on the findings? | p. 6, Section 2.5: formal participant/member checking was not conducted. |  |  |  |
|  | **Reporting** | | | |  |  |
|  | 29. Quotations presented | Were participant quotations presented to illustrate the themes/findings? Was each quotation identified? e.g., participant number | p. 8-10, Sections 3.3-3.7: quotations presented and identified by participant code. |  |  |  |
|  | 30. Data and findings consistent | Was there consistency between the data presented and the findings? | p. 8-10 and Supplementary Table S1: findings are supported by quotations and thematic synthesis. |  |  |  |
|  | 31. Clarity of major themes | Were major themes clearly presented in the findings? | p. 8, Table 3 and Sections 3.3-3.7: five major themes clearly presented. |  |  |  |
|  | 32. Clarity of minor themes | Is there a description of diverse cases or a discussion of minor themes? | p. 8, Table 3 and Sections 3.3-3.7: subthemes and diverse/challenging cases described. |  |  |  |

**Table S2.** Transcription of phenomenological interviews

| Participant | Question | Italian Response | English Translation |
| --- | --- | --- | --- |
| P1 | 1 | Adesso sono più in forza mentre prima ero più debole | Now I am stronger, whereas before I was weaker |
| P1 | 2 | Il Case Manager è una persona speciale. Non una in particolare, ma è gentile, risponde sempre se hai bisogno | The Case Manager is a special person. Not one in particular, but she is kind and always responds when you need her |
| P2 | 1 | Bene, non mi trovo ancora a mettere il mastice, ma per il resto mi sono ripreso bene | Well, I am not yet able to apply the adhesive, but otherwise I have recovered well |
| P2 | 2 | Mi sono trovato bene. Non una in particolare, ma mi ha aiutato molto come supporto morale | I felt good. Not one in particular, but she helped me a lot as moral support |
| P3 | 1 | Adesso sto abbastanza bene, attendo il prossimo controllo urologico. | Now I am feeling quite well, I am waiting for my next urological check-up. |
| P3 | 2 | Assolutamente bene, il CM è un personaggio straordinario che se ce l’avete, tenetevelo caro. La figura del Case Manager mi è stata utile perché se dovessi avere problemi, la prima persona che contatto è lei. | Absolutely well, the CM is an extraordinary person, if you have one, keep them close. The Case Manager was helpful because if I had any problems, she was the first person I contacted. |
| P4 | 1 | Bene, sto bene. Mi sono ripreso bene e ho imparato a conviverci con il sacchetto. | I am well. I have recovered well and learned to live with the bag. |
| P4 | 2 | È una persona attiva, ti chiede, ti da consigli, ti tira su di morale. Un po’ un riferimento | She is an active person, she asks questions, gives advice, and lifts your spirits. A bit of a reference point. |
| P5 | 1 | Non sono quello di prima, ma ho la forza di volontà di andare avanti. Diciamo che il mio stato di salute è migliorato. | I am not the same as before, but I have the willpower to move forward. My health has improved. |
| P5 | 2 | Il Case Manager è una figura molto importante perché viene vista come una figura che ti sta proteggendo durante il percorso. | The Case Manager is a very important figure because she is seen as someone who protects you during the process. |
| P6 | 1 | Un pochino meglio dal punto di vista fisico | A little better physically |
| P6 | 2 | Figura eccellente, il mio riferimento. Mi è stata tanto d’aiuto su tutto. | An excellent figure, my reference. She helped me a lot with everything. |
| P7 | 1 | Fisicamente sto bene, ma non accetto ancora i sacchetti. | Physically I am fine, but I still don't accept the bags. |
| P7 | 2 | Mi sono trovata benissimo, è sempre gentile, carina, mi telefona sempre per sapere come sto. | I had a great experience; she is always kind, caring, and calls me to check on me. |
| P8 | 1 | Abbastanza bene, è complesso, ma potrebbe andare peggio. | Quite well, it's complex, but it could be worse. |
| P8 | 2 | Meglio di così era impossibile. È stata un’assoluta Stella Polare. | Better than this was impossible. She was an absolute guiding star. |
| P9 | 1 | Mi auguro che continui così, sto bene. Ho ripreso la mia vita di prima. | I hope it continues this way; I feel good. I have resumed my life. |
| P9 | 2 | Il Case Manager è una persona squisita, preparata. | The Case Manager is an exquisite and well-prepared person. |
| P10 | 1 | Discretamente bene, unico trauma che mi rimane dopo l’intervento è l’attività intestinale. | Fairly well, the only trauma that remains after surgery is intestinal activity. |
| P10 | 2 | Con Il Case Manager tutto bene: avere una figura che ti accompagna a livello di assistenza nel mondo chirurgico. | Everything went well with the Case Manager: having a figure who supports you in the surgical world. |
| P11 | 1 | Sto leggermente meglio, ho avuto un periodo in cui l’intestino non si era ancora stabilizzato. | I feel slightly better, I had a period when my intestines were not yet stable. |
| P11 | 2 | È veramente brava: velocissima e precisissima: un gioiello. | She is really great: very fast and precise, a jewel. |
| P12 | 1 | Sto bene, mi sono ripreso bene, avevo perso 10kg dopo l’intervento. | I am well, I have recovered well, I lost 10kg after the surgery. |
| P12 | 2 | Con Il Case Manager mi sono trovato benissimo, è sempre disponibile. | I got along very well with the Case Manager, she is always available. |
| P13 | 1 | Sto meglio, però mi è spuntato di recente un laparocele. | I feel better, but recently a laparocele has appeared. |
| P13 | 2 | Con Il Case Manager mi sono trovato benissimo, non mi posso lamentare. | I got along very well with the Case Manager, I can't complain. |
| P14 | 1 | Sto abbastanza bene grazie, mi sono ripreso bene. | I feel quite well, thanks, I have recovered well. |
| P14 | 2 | Con Il Case Manager mi sono trovato benissimo, è stata molto utile. | I got along very well with the Case Manager, she was very helpful. |
| P15 | 1 | Mi sento bene. Mi sono ripresa alla grande. | I feel good. I have recovered very well. |
| P15 | 2 | Mi sono trovata molto bene, una persona molto disponibile, empatica. | I felt very well, a very helpful, empathetic person. |
| P16 | 1 | Sto molto bene, mi sono ripreso bene. | I feel very well, I have recovered well. |
| P16 | 2 | Il Case Manager mi ha seguito bene, mi ha spiegato sempre tutto. | The Case Manager followed me well, always explained everything. |
